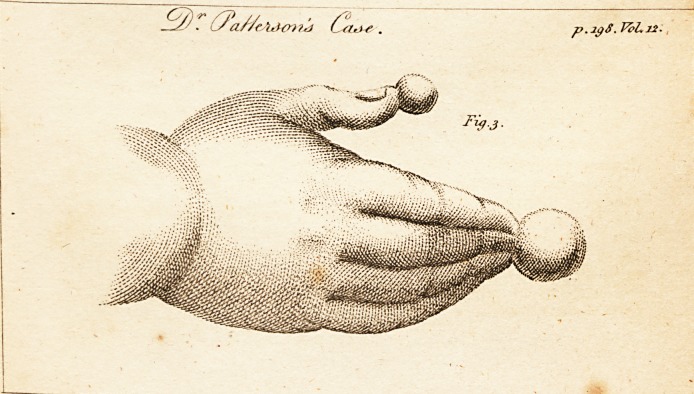# Case of Brainular Affection from an External Cause

**Published:** 1804-09-01

**Authors:** 

**Affiliations:** Londonderry


### Case of Brainular Affection from an External Cause; *by* Dr. Patterson, *of Londonderry.*

MONDAY, Nov. 15th, 1802, near noon, I was called to visit Master W— M—, a strong healthy boy, aged four years; but previous to my introduction into the chamber where he lay, I received the following information from his mother. On the preceding Saturday, in the afternoon, she observed him playing with an iron stair-rod, which she permitted *him* to keep, and left him at his play with some others of her children, not having the most remote idea that any mischief could ensue from his handling that moveable. However, she had not long left the spot when he came to her with his nose bleeding, which she was told proceeded from the rod having accidentally ran up his left nostril, whence it was recently withdrawn. The rod is nearly cylindrical, and not more than the sixth part of an inch in diameter at the ends.

He did not complain of pain; neither was any point in the whole course of the nose, nor in the neighbouring parts, painful on pressure with the finger; but shortly after the mischance, he began to vomit, and was put to bed. Before I saw him, a purge or two of calomel had been given; a blistering plaster was applied to the back; and other corresponding measures were pursued, on the presumption, it seems, that the morbid appearances proceeded from worms.

I found him lying in rather a drowsy state, not qualified to make a direct or satisfactory answer, but seeming sensible when roused by importuning him with questions.

His

His eyes did not betray any irregularity in their motions, indistinctness of vision, nor diseased condition in the pupils. On being raised in the bed, he indicated a propensity to vomit, by small sharp regurgitating motions of the œsophagus; and when taking drink, he swallowed it in a hurried manner. His face, which was flushed, shewed a degree of turgescence; and, shortly before I saw him, some twitchings about the alæ nasi and lip were observed, then, however, scarcely discernible. His pulse, at the same time, was too unequal to be accurately numbered; but it communicated to the fingers a sense of considerable acceleration.

Considering the grounds upon which the previous treatment had been instituted, reflecting on the phenomena before my eyes, and never having met with a similar case, I found myself in a sort of dilemma; therefore I thought it most expedient to commence my proceedings with caution. Accordingly, I ordered frequent doses of citrated kali, diluting fluids, and cool air. In the evening, the drowsy and other symptoms continued; but the pulse became quite uniform, and so distinct that it could be accurately counted, by which it was found to be from 108 to 110 beats in a minute, Under these circumstances, I thought nothing requisite, during the night, except vin. antim. a few drops of which were directed to be given every second hour.

Next morning, a brainular affection became apparent, as indicated by increased stupor, more extensive twitchings, and very indistinct pulse; whilst the iris still retained tolerable sensibility, which it did not lose until an advanced stage of the malady. Convinced now that my original suspicions of the real nature of the case were well founded, I prescribed a blistering plaster to the head, leeches to the temples, laxatives, and enemas. The leeches and injections, with some minor expedients, were repeatedly employed; but the morbid phenomena hourly increased, until convulsions closed the scene about twelve o'clock on Wednesday night, concluding the space of four days and eight or ten hours from the occurrence of the accident.

Arguments and solicitations were used to obtain leave to make an anatomical examination, but in vain; a prejudice prevailed, whose influence, in this case, was particularly to be regretted, as I consider it a very rare casualty, not having met with a like instance in the course of my reading, nor even heard of any, except one, which happened in England. That also took place in a child, and terminated fatally in a shorter time than the one here recorded; but of the attending appearances I could not get an account in the least satisfactory, nor was the case, I believe, communicated in any shape to the public.

From a contemplation of the above circumstances, it seems fair to infer, that the instrument penetrated the brain, and that it passed through the cerebral, and toward the orbitar, plate of the ethmoid bone; for I cannot conceive upon what principle or analogy the symptoms and fatality could occur from a superficial injury done to the olfactory nerves, the recurrent portion of the nasal, and the sphæno-palatine twig, as they are distributed on the pituitary membrane of the nose. However, I would be glad to know, Are such injuries as this patient suffered medicable wounds, or are they past all Surgery?

*July* 7, 1804.

P.S. The enclosed drawing *(see the plate)* is the figure of a hand belonging to a child, about a week old, who was brought to me in 1795. As represented in the figure, the tops of the thumb and fingers were surmounted with knobs, of a gristly or cartilaginous nature; and the fingers, united at the points by one of these knobs, were also bound together with membranes. The knobs I cut off, and severed the fingers, both which were easily done, and followed by complete success. As the case appears rare, I request its insertion in your Journal, which will make it universally known.

## Figures and Tables

**Fig.3. f1:**